# Lung Cancer with Skin and Breast Metastasis: A Case Report and Literature Review

**DOI:** 10.1155/2015/136970

**Published:** 2015-03-16

**Authors:** Bikash Bhattarai, Marie Frances Schmidt, Meenakshi Ghosh, Abhisekh Sinha Ray, Saveena Manhas, Vikram Oke, Chidozie Charles Agu, Md. Rawshan Basunia, Danilo Enriquez, Joseph Quist, Catherine Bianchi, Ravi Hans, Saroj Kandel

**Affiliations:** Department of Medicine, Interfaith Medical Center, 1545 Atlantic Avenue, Brooklyn, NY 11213, USA

## Abstract

Lung cancer is one of the most common cancers in America. Frequent sites of metastasis include the Hilar lymph nodes, adrenal glands, liver, brain, and bone. The following case report is of a primary lung cancer with metastases to the breast and skin. *Case*. A 48-year-old African American male with a past medical history of poorly differentiated left breast cancer status after modified radical mastectomy (MRM), chronic obstructive pulmonary disease, and smoking (20 pack-years) presents to the ER with progressive shortness of breath on exertion, upper back pain, and weight loss for 2 months in duration. On physical examination he is found to have a MRM scar on his left breast and a left periumbilical cutaneous mass. Chest X-ray and chest CT reveal a right upper lobe mass and biopsies from the breast, lung, and the periumbilical mass indicate a poorly differentiated carcinoma of unclear etiology; all tumor markers are negative. The patient is male and a chronic smoker; therefore the diagnosis is made as lung carcinoma with metastases to the breast and skin. *Conclusion*. A high index of suspicion for cutaneous metastases should be cast when investigating cutaneous pathologies in patients at risk for primary lung malignancy.

## 1. Introduction

Every year thousands of Americans across the country are diagnosed with lung cancer and for the majority of these patients the diagnosis is terminal. Lung cancer is among the most common cancers worldwide. In the United States and other industrialized countries it is the major cause of cancer mortality, primarily because of exposure to cigarette smoke. Lung cancer is the leading cause of cancer deaths worldwide in men and is second most common in women. In 2012, lung cancer occurred in approximately 1.8 million patients and caused an estimated 1.6 million deaths [1]. In the United States, lung cancer will occur in an estimated 224,000 patients and cause about 159,000 deaths [2]. The prognosis of lung cancer is poor with a 5-year survival rate standing at approximately 15%. The most common histologic type is adenocarcinoma, followed by squamous cell carcinoma, small cell carcinoma, and large cell carcinoma [3]. Frequent sites of metastasis often include the hilar lymph nodes, adrenal glands, liver, brain, and bone, with rare cases metastasizing to the skin [4]. Although rare, these cutaneous metastases are an important diagnostic clue since primary lung lesions are often quiescent making the initial diagnosis of lung cancer very difficult. In general, cutaneous lesions as an initial manifestation of internal neoplasia represent only 0.8% of total cases but if they are present, they imply a very advanced grade of disease and a very poor prognosis [5].

## 2. Case History

**Figure 1:**
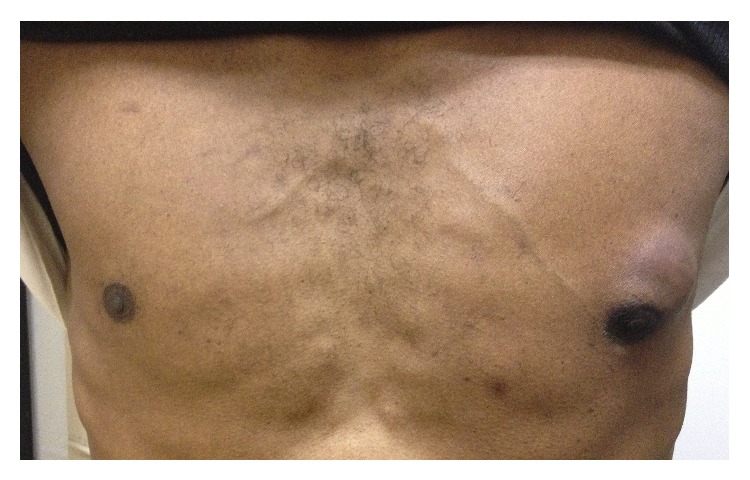
Subcutaneous mass invading left breast tissue (from previous admission).

A 48-year-old African American male with a past medical history of infiltrating poorly differentiated left breast cancer (Figure 1) without lymph node involvement s/p modified radical mastectomy (MRM) along with axillary dissection 2 months earlier, COPD, bipolar disorder, and chronic smoking (20 pack-years) presented to the ER with progressive SOB for the past 2 months. The patient complained of being dyspneic on minimal exertion and also complained of upper back pain for the last 2 months which was progressive, 10/10 in intensity, sharp in quality, nonradiating, aggravated with exertion, and alleviated with supine posture and analgesic medication (ibuprofen). The pain was not associated with any sensory or motor symptoms and the patient denied tingling, weakness, or numbness of the lower extremities, bowel, and bladder incontinence. He gave a history of approximately 30 lb. weight loss associated with anorexia for the past 6 months but denied fever, cough, chills, chest pain, nausea, vomiting, or any other systemic complaints. He had no known drug allergies and was on home medications of Ibuprofen, Trazodone, Citalopram, Albuterol, and Tiotropium inhaler. He was an active smoker and admitted to using alcohol and marijuana occasionally. His family history was positive only for colon cancer in his mother.

**Figure 2:**
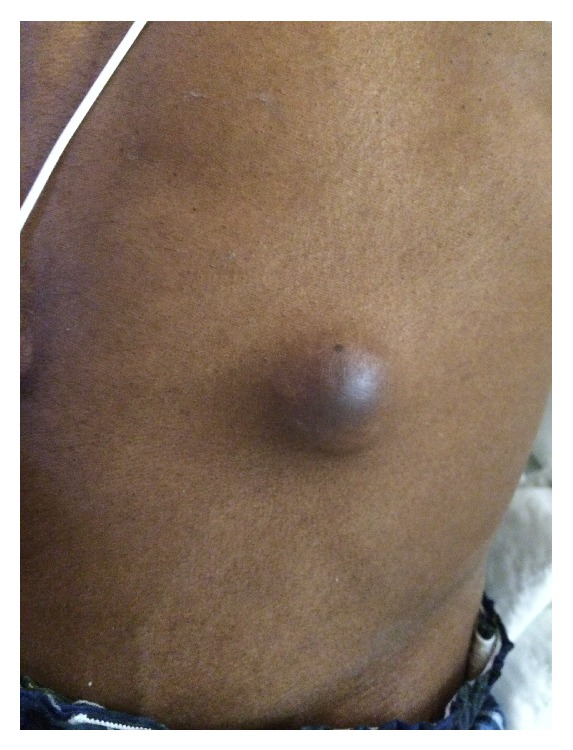
Left abdominal wall mass.

On admission, the patient was tachycardic (pulse 105/min) and tachypneic (respiratory rate 20) but was saturating at 100% on room air. He was afebrile and normotensive. On general examination, he appeared cachectic and in moderate respiratory distress. The head was atraumatic and normocephalic; pupils were bilaterally equal and reactive. There was no evidence of neck vein distension or pedal edema. A well-healed surgical scar of MRM was noted extending from his left axilla to his sternum. On percussion, the right chest appeared to be dull and on auscultation breath sounds were decreased in the right base in comparison to the left. There was a soft, nontender mobile mass in the periumbilical area which was 2 cm × 2 cm × 2 cm in size and appeared to be superficial on examination (Figure 2). Other than the mass, the abdomen was soft, nontender, and nondistended with normoactive bowel sounds. Tenderness was elicited on palpation of the upper thoracic spine and right scapular area and range of motion of both arms was mildly restricted due to pain and discomfort. Cardiovascular and neurological examination was noncontributory.

**Figure 3:**
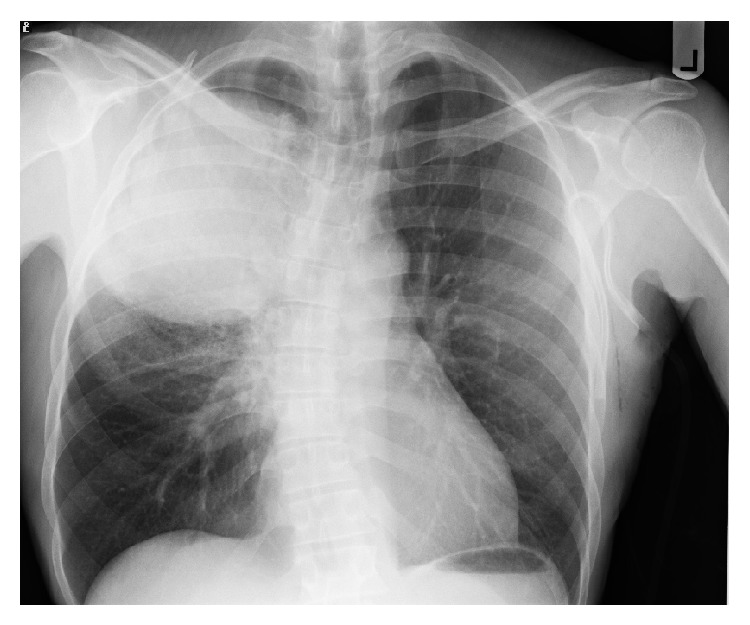
Chest X-ray: Rt upper lobe mass.

On laboratory examination, the patient was found to have normocytic normochromic anemia with hemoglobin of 9 gm/dL and thrombocytosis (platelet count of 713). The leukocyte count was normal. Metabolic panel and liver function test results were within normal limits except that there was a mildly elevated alkaline phosphatase of 125. The coagulation panel was normal. The chest X-ray was reported as a deviation of the tracheal lumen to the left without obstruction and a right upper hemithorax mass (Figure 3).

**Figure 4:**
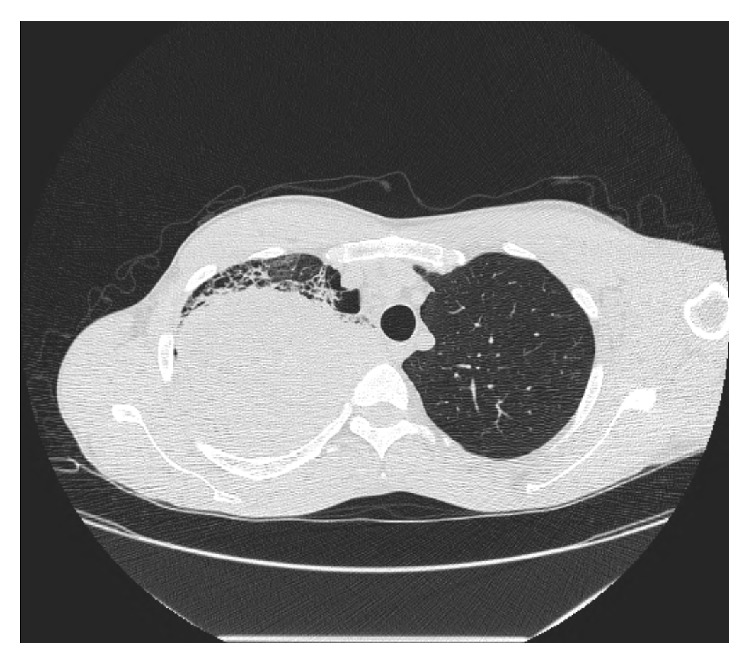
Chest CT scan: Rt upper lobe density.

**Figure 5:**
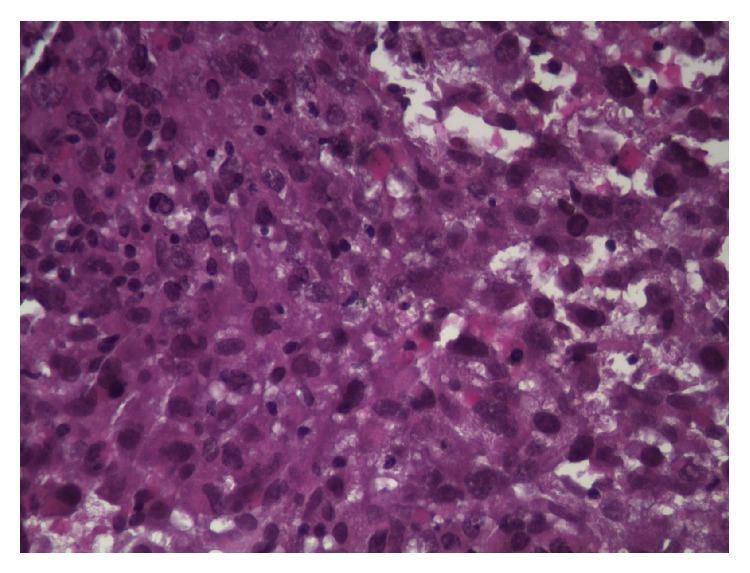
Lung transbronchial biopsy specimen: poorly differentiated cluster of neoplastic cells with abundant cytoplasm with hyperchromatic nuclei. No gland or sheets of squamous cell formation.

**Figure 6:**
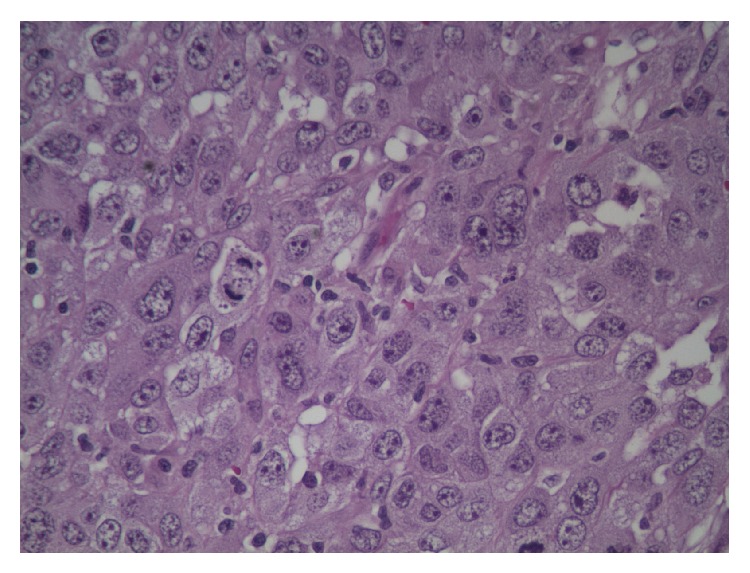
Abdominal wall biopsy specimen: poorly differentiated neoplastic cells with abundant cytoplasm with hyperchromatic nuclei. No gland or sheets of squamous cell formation.

The patient was admitted to the medical floor and work-up was done. CT scan reported a large right upper lobe lung mass suspicious for malignancy, bullous emphysematous disease with pulmonary fibrosis, and mediastinal lymphadenopathy with small pleural effusion on the right (Figure 4). CT scan of the thoracolumbar spine did not reveal any osseous erosions or lytic lesions but bone scan showed a small focus of intake uptake in the right scapular region. PET scan showed hypermetabolic activities in the right lung, mediastinum, and the subcutaneous abdominal nodule. The patient underwent biopsy of the right upper lobe lung mass and abdominal mass. Biopsy results of the abdominal mass came back as high-grade malignant neoplasm with unclear etiology whereas the lung mass biopsy was reported as poorly differentiated non-small-cell lung cancer (Figures 5 and 6).

**Figure 7:**
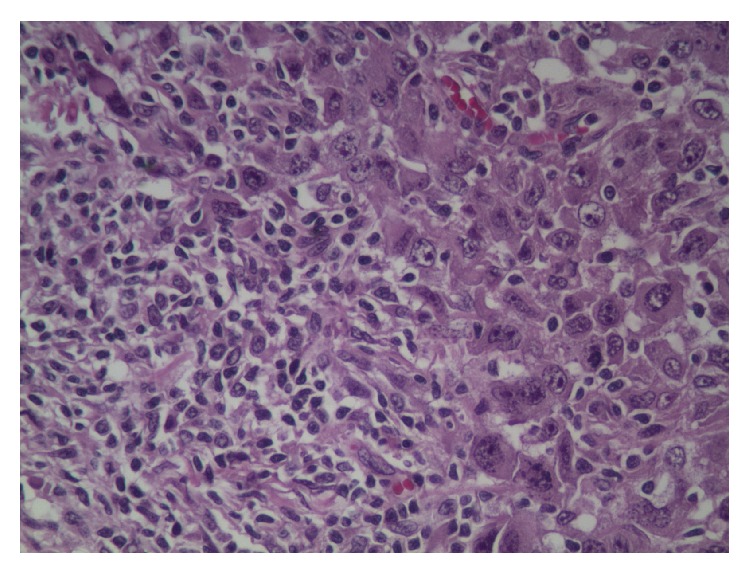
Left breast tissue (high power): poorly differentiated malignant cells, prominent nucleoli.

**Figure 8:**
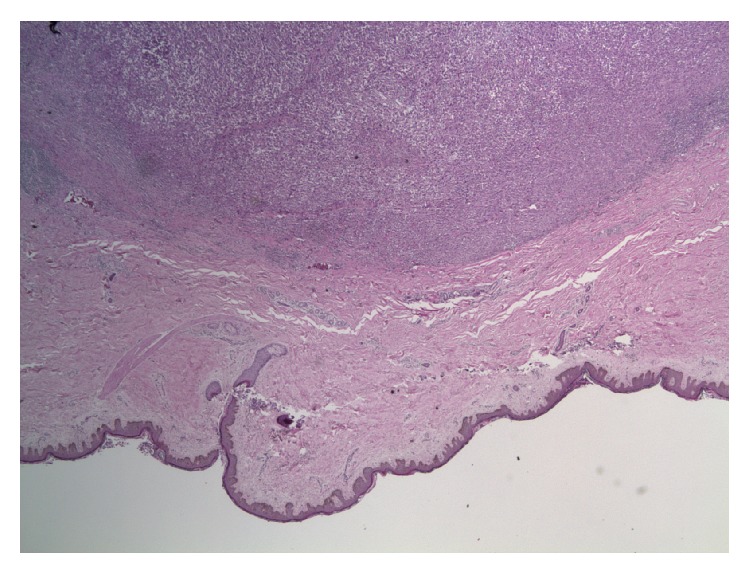
Infiltrating poorly differentiated carcinoma and surgical margin free of tumor. No ductal and no parenchymal tissues identified.

**Figure 9:**
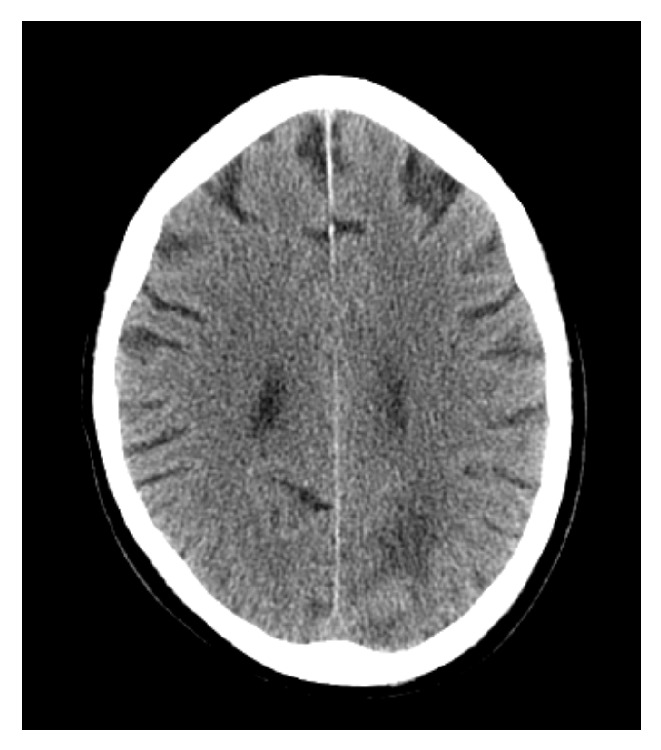
Head CT scan showing multiple brain metastases left breast tissue (low power field).

Retrospectively, upon reviewing the presurgical chest X-ray there was a right upper lobe mass that was not described before breast surgery. The markers for breast and lung cancer (ER, PR, HER2, GCDPF-15, Mammaglobin, BCA225, GATA3, anti-TTF, CK7/20, and napsin A) were negative in all biopsies taken (breast (Figures 7 and 8), lung, and abdominal wall); however since the patient was male and a chronic smoker the diagnosis was made as Stage IV non-small-cell lung cancer with soft tissue metastases to both the abdominal wall and breast. Furthermore, head CT indicated multiple brain metastases (Figure 9).

As the histopathology from all three biopsy sites revealed poorly differentiated cancer with metastases and all tumor markers to be negative, the oncology team recommended palliative chemotherapy for non-small-cell carcinoma of lung. The patient was treated with carboplatin and paclitaxel for 3 cycles. After the 3rd chemotherapy cycle he developed brain metastases and passed away 11 months after the primary diagnosis.

## 3. Discussion

In this case report a patient with an erroneous diagnosis of primary male breast cancer s/p MRM with radical axillary dissection is in actuality found to have non-small-cell lung cancer with cutaneous metastases to both the breast and abdomen. Around 80 to 90 percent of all cutaneous metastases in adults are due to malignancies originating from internal organs such as the lungs, breasts, melanoma, oral cavity, colon, kidney, ovary, or stomach [4, 6, 7]. However, there are notable differences in gender and cutaneous metastases in men are mainly due to primary lung malignancies (12%–28%), GI malignancies (11–19%), and melanomas (13%–32%) whereas in women the lungs are the fifth most common primary site (4%) after the breast (69%), large intestine (9%), melanoma (5%), and ovaries (4%). The percentage of patients with primary lung cancer that develop cutaneous metastases ranges from 1 to 12 percent, and in a large series the skin was only the 13th most common site for metastasis from the lung [4, 8]. Although lung cancer does not metastasize to the skin often, when it does metastasize it does so very quickly with 5.75 months being the average time for a primary lung cancer to present with skin lesions [9] and in 20 to 60 percent of cases the skin lesions are present before or alongside the diagnosis of the primary tumor [6, 10, 11]. Although quite uncommon, skin lesions are very important in the workup of patients presenting with a history of tobacco use [12] since they may be the first sign of a primary lung malignancy or even of a recurrence [6].

Our case illustrates a lung cancer which has metastasized both to the breast and the abdomen; however, this is quite rare; instead, most lung cancers usually involve the anterior chest, abdomen, and head and neck [11, 13–15] while less common locations include the shoulder, flank, and lower and upper extremities [11, 13, 14]. Rare sites of metastasis include the gingiva, scrotum, perianal skin, lip, nose, burn scars, fingers, and toes [11, 16, 17]. Cutaneous metastases from lung cancer do not have a characteristic presentation and are often described as nodular, mobile or fixed, hard or flexible, single or multiple, and painless. Furthermore, less commonly, these lesions may also present as either papular, plaque like, ulcerated, vascular, zosteriform, erysipelas type, or lastly scarring alopecia on the scalp [13, 18, 19]. The colors of the lesions vary from flesh colored to red, pink, purple, or bluish black and the sizes vary from 2 mm to 6 cm in diameter [12]. Furthermore, cutaneous metastases from the lung are frequently poorly differentiated [12, 20] and they may typically invade the lymphovascular system but are usually limited to the dermis and subcutaneous layers of skin [21]. The most common lung cancer to present with cutaneous manifestations is adenocarcinoma, followed by squamous cell carcinoma or small cell carcinoma and then large cell carcinoma (LCC) [11, 13, 20–22].

Histopathology is the best method to correctly classify cutaneous lesions; however, in addition to histology, immunohistochemical markers may be useful classification tools when the primary site of the malignancy is unknown and both the clinical and histological information are inconclusive [21, 23]. Furthermore, two markers that may be useful in cases where the primary malignancy is unknown include antithyroid transcription factor (TTF) and CK7/20. Anti-TTF is a marker that is both sensitive and specific for primary adenocarcinomas, bronchoalveolar carcinomas, and small cell carcinomas when a thyroid primary is ruled out [24], whereas CK7+/20 is a marker that is sensitive but not specific for primary adenocarcinomas and bronchoalveolar carcinomas [24–26].

The treatment of solitary cutaneous masses is either surgery alone or surgery combined with radiation and or chemotherapy [27, 28]. It has been proposed that surgery may increase survival in patients with solitary cutaneous masses and that patients who receive surgical treatment survive an average of 12.5 months after diagnosis [27–29]. This is in contrast to the shorter survival of 6.5 to 8 months of patients who receive chemotherapy alone. However, despite the shorter survival, if there are multiple cutaneous lesions or internal metastases, then chemotherapy is the primary option and the response of the skin lesions to chemotherapy may actually be used to monitor the response of the internal malignancy. Similarly, radiation can be effectively used alone and/or in combination with both chemotherapy and surgery. However, radiation is usually not very effective and instead is used as a palliative measure in lesions that are either painful or bleeding [12, 20, 22, 29, 30].

More recently improved understanding of the pathobiology of non-small-cell lung cancer (NSCLC) has led to the development of small molecules that target genetic mutations known to play critical roles in the progression to metastatic disease. Mutations in epidermal growth factor receptor (*EGFR*),* KRAS*, and anaplastic lymphoma kinase (*ALK*) are mutually exclusive in patients with NSCLC, and the presence of one mutation in lieu of another can influence response to targeted therapy. Therefore, testing for these mutations and tailoring therapy accordingly is widely accepted as standard practice. Use of the EGFR-TKIs erlotinib and afatinib is limited to patients with adenocarcinomas who have known activating* EGFR* mutations [9, 31, 32]. Because* EGFR* and* ALK* mutations are mutually exclusive, patients with* ALK* rearrangements are not thought to benefit from EGFR-targeting TKIs. Instead, treatment with an ALK inhibitor (crizotinib, ceritinib) is indicated [33–35]. Also crizotinib has shown potent antitumor activity in a second subgroup of patients with non-small-cell lung cancer (NSCLC)—those with advanced ROS1 protooncogene receptor tyrosine kinase- (*ROS1*-) rearranged tumors, which are found in about 1% of NSCLC patient adenocarcinomas [36].

Cutaneous metastases associated with primary lung cancer suggest a poor prognosis and some poor prognostic indicators include multiple cutaneous metastases, nonresectable or small cell primary tumors, or other distant metastases [28]. Survival rates vary with each individual case; however, patients initially presenting with cutaneous metastases live approximately 3-4 months less than patients who develop cutaneous metastases later in their disease process. Furthermore, after the diagnosis of a cutaneous metastasis the mean survival is usually about 5-6 months, but some patients may survive for longer than one year [12, 21, 22, 30]. In our case our patient died within 11 months of the primary diagnosis.

## 4. Conclusion

In this case study we discuss a patient with a 20 pack-year history of smoking who initially presented with skin manifestations suggesting primary breast cancer in a male which upon further investigation were diagnosed as the cutaneous manifestations of a non-small-cell lung cancer. A high index of suspicion should always be present when cutaneous pathology is seen in a patient with a clinical history suggestive of primary lung cancer. If cutaneous metastases are suspected the prognosis is extremely unfavorable and the use of appropriate tests like histology from a skin biopsy, immunohistochemical stains and/or electron microscopy should always be pursued. Cutaneous metastases embark a poor prognostic feature and although treatment is available it does not improve survival significantly.
